# The Effects of a 16-Week School-Based Exercise Program on Anxiety in Children with Autism Spectrum Disorder

**DOI:** 10.3390/ijerph19095471

**Published:** 2022-04-30

**Authors:** Marie Carey, Damien Sheehan, Sean Healy, Fiona Knott, Sharon Kinsella

**Affiliations:** 1 Autism Research Group, Department of Health and Sport Science, Institute of Technology Carlow, R93 V960 Carlow, Ireland; mariec1995@gmail.com (M.C.); damien.sheehan@itcarlow.ie (D.S.); 2 School of Nursing, Psychotherapy and Community Health, Dublin City University, D09 Y5N0 Dublin, Ireland; sean.d.healy@dcu.ie; 3 Department of Psychology, University of Reading, Reading RG6 6EU, UK; f.j.knott@reading.ac.uk

**Keywords:** autism, physical activity, anxiety, home, school, exercise

## Abstract

Physical activity interventions have been shown to decrease anxiety in children with ASD. There is little known regarding the effects of an exercise program on anxiety in both home and school settings and the optimal dosage to reduce anxiety. Therefore, the aim of this study was to assess the effects of a 16-week exercise program on the anxiety levels of children with moderate to severe symptoms of ASD in home and school settings, and to compare the effects at 8 and 16 weeks. This study was a within-subject, non-controlled design, intervention study. Twenty-four children (5–18 years) with moderate to severe ASD were included. A school-based exercise program was implemented three days a week for 16 weeks. Parents and teachers completed the Anxiety Scale for Children for ASD (ASC-ASD) at baseline, week 8, and week 16. A one-way repeated-measure ANOVA with post hoc analysis using Bonferroni adjustment was used to test for a significant effect for time (*p* < 0.05), with Cohen’s *d* used to calculate the effect size. For teacher-reported anxiety, there were significant decreases from baseline to week 16 for total ASC-ASD (*p* < 0.001), performance anxiety (*p* < 0.001), anxious arousal (*p* < 0.001), and uncertainty (*p* < 0.001). There was no significant decrease in parent-reported anxiety. The findings demonstrate that a 16-week exercise program can reduce anxiety in children with ASD in school settings. Results demonstrate that 16 weeks, as opposed to 8, may be necessary to have a significant effect on in-school anxiety.

## 1. Introduction

Anxiety is a common comorbidity among children with autism spectrum disorder (ASD), with up to 80% of children with ASD meeting the diagnostic criteria for at least one anxiety disorder [1,2,3]. Children with ASD most commonly present with specific phobia disorder, obsessive-compulsive disorder, social anxiety disorder, and separation anxiety [1,4]. Anxiety comorbidity in children with ASD can be associated with social avoidance, difficulties establishing relationships, mood problems, and disruptive behavior [5,6,7,8]. These behaviors can occur at home and in school settings and can have a significant impact on their quality of life [9].

In response to the prevalence and debilitating effects of anxiety for children with ASD, there has been increased focus on identifying effective treatment options for this population [10,11]. Pharmacological interventions are a common treatment for anxiety in children with ASD [12]. However, there is limited evidence to support the effectiveness of psychotropic medication to treat anxiety in children with ASD [13,14]. Additionally, a high rate of adverse effects has been associated with the consumption of this type of medication. Such side effects include aggression, agitation, insomnia, tics, headaches, and depression [15]. Psychotropic medication has also been shown to lead to significant weight gain and an increased appetite in children with ASD [16]. Another common treatment approach for anxiety is cognitive behavioral therapy (CBT), which has been shown to reduce anxiety in children with mild to moderate symptoms of ASD [17]. As CBT is a cognitively and verbally demanding treatment technique, the generalizability of its effects for children with more severe symptoms of ASD is questionable [18]. Children with moderate to severe symptoms of ASD may be unable to express emotions and identify anxious stimuli, and therefore may not experience the benefits of CBT. Given this challenge, there are inadequate treatment options for children with more severe symptoms of ASD that suffer from anxiety.

Exercise interventions have previously been shown to reduce certain anxiety disorders in neurotypical adolescents and adults, including separation anxiety, generalized anxiety disorder, panic disorder, agoraphobia, and post-traumatic stress disorder [19,20,21,22]. Furthermore, exercise can produce similar anxiety-reducing effects to CBT or medication [21]. Hence, exercise may be a possible treatment for anxiety in children with ASD. A limited number of studies have supported the anxiolytic effect of physical activity in children with ASD. Hillier et al. [23] implemented an 8-week low-intensity physical activity program once a week with 18 adolescents and young adults (13–27 years) with high-functioning ASD. The intervention consisted of aerobic, flexibility, balance, and muscular strengthening exercises. This program led to a significant within-session reduction in self-reported anxiety and cortisol levels. However, significant reductions were not maintained over the 8 weeks. In a more recent study, Golsefidi and Hashemi [24] implemented a 4-week motor program three times a week with 10 children (6–10 years) with Asperger syndrome and demonstrated a significant reduction in their self-reported anxiety levels. Therefore, physical activity may be a cost-effective and non-invasive treatment for anxiety in children with ASD.

The exact mechanism responsible for the reduction in anxiety following physical activity is unclear. There are several physiological adaptations following exercise proposed in neurotypical individuals [25]. It is possible that some of these adaptations may also occur in children with ASD. In brief, some of the biological adaptations following exercise include a reduction in anxiety-producing hormones (such as cortisol and glucocorticoids) and improved synthesis of neurotransmitters (such as serotonin and noradrenaline) and opioids (β-endorphins) [26,27,28]. These adaptations may play a crucial role in explaining the reduction in anxiety symptomology associated with exercise participation [29,30,31]. Exercise may also lead to psychological adaptation, such as increased self-efficacy, decreased anxiety sensitivity due to repeated exposure to physiological sensations associated with anxiety, and as an element of distraction [32,33,34].

Hillier et al. [23] and Golsefidi and Hashemi [24], albeit limited, demonstrate that physical activity interventions have the potential to reduce anxiety in children with ASD. However, three critical gaps in the literature remain. Previous studies have been conducted with high-functioning children with ASD, excluding those with more moderate or severe ASD. It is unknown if exercise has similar anxiety-reducing effects in children with moderate to severe symptoms of ASD. This shortcoming requires investigation as children with more severe symptoms of ASD may experience similar or greater levels of anxiety [4,35]. The prior research on physical activity interventions for children with ASD to reduce anxiety was of a short duration (e.g., 4 weeks [24]; 8 weeks [23]). Studies of the effects of longer-duration interventions are needed to identify an optimal dosage. Finally, research to date has examined self-reports of anxiety. Data demonstrate that anxiety is particularly prevalent and detrimental for children with ASD in school settings [36]; therefore, it is necessary to examine the effects of exercise interventions on children with ASD in both home and school settings.

To begin to address these gaps in the literature, the purpose of this pilot study was twofold: (a) to assess the effects of a 16-week school-based exercise program on the anxiety levels of children with moderate to severe symptoms of ASD in home (i.e., parent-reported) and school settings (i.e., teacher-reported), and (b) to compare the effects of an exercise intervention on anxiety levels at 8 and 16 weeks.

## 2. Materials and Methods

### 2.1. Participants

Participants were recruited from two local schools, and classes within two mainstream schools, that specialized in the education of students with ASD. Informational leaflets were sent home with information for parents on the purpose of the study. Children whose parents were interested in participating in the study were screened for the following inclusion criteria: (a) aged between 5 and 18 years of age; (b) had received a diagnosis of ASD by a psychologist; and (c) had a moderate to severe level of ASD, as measured by the Gilliam Autism Rating Scale—2nd Edition (GARS). Moderate ASD was defined as having a raw GARS score of 10–33 and severe ASD severity was defined as having a raw GARS score of ≥34 [37].

Children were excluded if: (a) they had not been diagnosed with ASD; (b) they had any injuries or medical conditions preventing them from participating in exercise; or (c) a family doctor advised against partaking in exercise. If a child was unable to give consent, parents/guardians gave consent on behalf of their child and completed a medical screening form. Ethical approval was granted by the Institutional Ethics Committee from the primary author’s institution.

**Figure 1:**
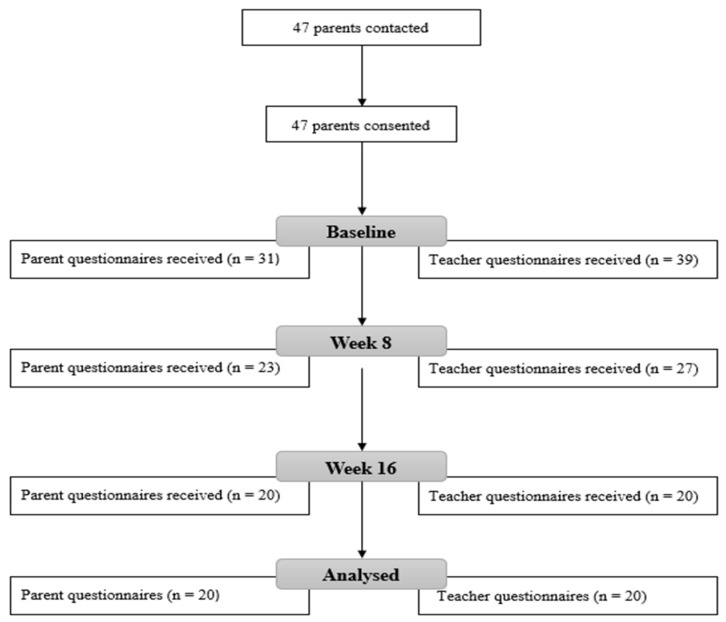
Process of gathering data.

Forty-seven participants were enrolled at the beginning of the study; however, completed data were only obtained from either parents or teachers on twenty-four children following 16 weeks of intervention (see Figure 1). Children’s data were excluded from the final analysis if: (a) they had an attendance of less than 70% over the 16 weeks; (b) data were not received for all three timepoints, for the child, for either the parent-reported or teacher-reported scales. Complete data on both parent- and teacher-reported scales were received for sixteen children. Four additional children had complete data on parent-reported scales but incomplete teacher-reported scales, giving a total of twenty participants in the parent-reported group. An additional four children had complete data on teacher-reported scales but incomplete parent-reported scales, giving a total of twenty participants in the teacher-reported group. Therefore, a total of twenty-four male children were included in the study.

### 2.2. Study Design

This study was a within-subject, non-controlled design, intervention study. The anxiety levels of the children were assessed at three timepoints during the study—at baseline, week 8, and week 16—using the Anxiety Scale for Children with Autism Spectrum Disorder (ASC-ASD) [38]. This scale was completed by parents, teachers, or both (see Figure 1).

### 2.3. Measures

At baseline, parents completed a questionnaire which queried their child’s age, gender, and interests so that the intervention could be adapted to suit the interests of participants. Height and weight were also measured at baseline using electronic weighing scales (Seca 807 Electronic Scale, Hamburg, Germany) and measuring tape (Seca 201 Limb Tape Measure, Hamburg, Germany).

The ASC-ASD was completed by parents and/or teachers to reflect the children’s anxiety symptoms present in the home and school settings. Teachers completed the forms while in school and parents completed the form at home.

The ASC-ASD is a recently developed scale specifically designed to assess anxiety symptoms in children with ASD [38]. It consists of four subscales, measuring performance anxiety, anxious arousal, separation anxiety, and uncertainty. Anxiety is rated on a 4-point scale from never (zero) to always (three). Two cut-off points for the ASC-ASD have been proposed by the authors of the scale: scores between 20 and 24 are categorized as significant anxious symptomatology, and scores > 24 are categorized as a more specific indication of significant anxiety [38].

The ASC-ASD has shown good to excellent reliability and validity for assessing anxiety in children with ASD. The scale has received good to excellent internal consistency (Cronbach’s α = 0.94) and excellent test–retest reliability (r = 0.82–0.84) [38]. The ASC-ASD has also shown moderate to strong correlations with the Spence Children’s Anxiety Scale (r = 0.66–0.88) and the Screen for Anxiety and Related Emotional Disorders (r = 0.88–0.91) [38,39].

### 2.4. Exercise Intervention

The 16-week exercise program was implemented three days a week for one hour per session. Each program was conducted in the participating children’s school sports hall, multi-activity room, or outdoor AstroTurf field, weather permitting. The one-hour session was divided into ten minutes for warm-up, forty minutes for the main phase, and ten minutes for stretches at the end. The program was implemented with groups of six to ten children at a time. The program was implemented by a health science graduate with one year of experience in delivering exercise programs to children with ASD. The participating children’s teachers and special needs assistants provided additional support.

The warm-up consisted of activities which aimed to gradually increase the children’s heart rate [40]. Movements incorporated into the warm-up included walking, running, jumping, and stretches to prepare the children for the main phase. The main phase focused on developing and progressing fundamental movement skills such as throwing, catching, jumping, landing, balancing, kicking, dodging, and striking with their hand or an implement. These movements were first isolated into simple tasks, which the children practiced with a teacher or special needs assistant, such as throwing and catching a ball. If a child was unable to complete activities independently with verbal instruction, the prompting hierarchy method was used [41]. A verbal prompt was first used, followed by a visual prompt, and then a model prompt. If necessary, a physical prompt was used if a child was unable to perform the task—for example, the teacher holding the child’s hands to guide the child to catch the ball.

Once the child was proficient at an activity, the level of difficulty was progressed. Lastly, activities were incorporated into group games involving all the children. An example of activities in the program can be seen in Table 1. Activities were adapted to suit the children’s abilities and interests. The cool-down consisted of a set of static stretches, which were held for approximately 30 s each [40].

### 2.5. Statistical Analysis

Data were assessed for normality using the Shapiro–Wilk test on IBM Statistical Package for Social Sciences (version 23). Significance level was set at 0.05. A one-way repeated-measure analysis of variance (ANOVA) was used to test for a significant effect for time, with effect sizes calculated using partial eta squared [42]. Partial eta squared was interpreted as 0.01 (small effect), 0.06 (moderate effect), and 0.14 (large effect) (Cohen, 1988). Post hoc analysis, with Bonferroni adjustment, was conducted to assess for significant differences between the three time points for normally distributed data. Cohen’s *d* was used to calculate the effect size of post hoc data, which was interpreted as <0.2 (trivial effect), 0.2–0.49 (small effect), 0.5–0.79 (moderate effect), and >0.8 (large effect) [43].

A Friedman test was used for nonparametric data and effect sizes calculated using Kendall’s *W* [43]. Kendall’s *W* was interpreted as <0.10 (small effect), 0.10–0.30 (moderate effect), and >0.30 (large effect) [44]. Post hoc analysis was conducted using pairwise comparisons with adjusted *p* values [45]. Pearson’s r was used to calculate effect sizes for post hoc data (Field, 2013). Pearson’s r was interpreted as 0.1–0.3 (small effect), 0.3–0.5 (moderate effect), and 0.5–1.0 (large effect) [43].

## 3. Results

Twenty-four children aged 5 to 18 years (M = 10.79, SD = 3.87) were included in the analysis. Six children met the diagnostic criteria for moderate ASD and 18 met the diagnostic criteria for severe ASD. Participants’ characteristics are presented in Table 2.

### 3.1. Teacher ASC-ASD

Twenty teachers completed the ASC-ASD at all three timepoints and were included in the analysis. A summary of results for teacher-reported ASC-ASD can be seen in Table 3. The Friedman test revealed a significant effect for time on total ASC-ASD scores, χ^2^(2) = 21.117, *p* < 0.001, *W* = 0.53. Post hoc analysis, with Bonferroni adjustment, revealed a significant decrease in total ASC-ASD scores by 12.25 points from baseline to week 16 (*p* < 0.001, *d* = 1.23). There were insignificant decreases from baseline to week 8 (*p* = 0.053, *d* = 0.36) and from week 8 to week 16 (*p* = 0.098, *d* = 0.8).

The Friedman test revealed a significant effect for time on the performance anxiety subscale of the ASC-ASD, χ^2^(2) = 20.863, *p* < 0.001, *W* = 0.52. Post hoc analysis, with Bonferroni adjustment, demonstrated a significant decrease in performance anxiety by 3.15 points from baseline to week 16 only (*p* = 0.001, *d* = 0.95). The Friedman test revealed a significant effect for time on anxious arousal, χ^2^(2) = 22.984, *p* < 0.001, *W* = 0.58. Post hoc analysis, with Bonferroni adjustment, showed a significant decrease in anxious arousal from baseline to week 8 by 1.1 points (*p* = 0.008, *d* = 0.47) and baseline to week 16 by 2.45 points (*p <* 0.001, *d* = 0.9). The one-way repeated-measures ANOVA revealed a significant effect for time on uncertainty, *F*(2, 38) = 15.285, *p* < 0.001, partial η^2^ = 0.45. Post hoc analysis, with Bonferroni adjustment, demonstrated a significant decrease in uncertainty from baseline to week 16 by 5.15 points (*p* < 0.001, *d* = 1.13) and week 8 to week 16 by 3.55 points (*p* = 0.008, *d* = 0.78). There was an insignificant effect for time on separation anxiety, χ^2^(2) = 3.6, *p* = 0.165, *W* = 0.09.

### 3.2. Parent ASC-ASD

Twenty parents completed the ASC-ASD at all three timepoints and were included in the analysis. A summary of results for parent-reported ASC-ASD can be seen in Table 4. Friedman tests revealed an insignificant effect for time on total ASC-ASD scores (χ^2^(2) = 0.187, *p* = 0.911, *W* = 0.01), performance anxiety (χ^2^(2) = 0.258, *p* = 0.879, *W* = 0.01), anxious arousal (χ^2^(2) = 1.782, *p* = 0.41, *W* = 0.05), and separation anxiety (χ^2^(2) = 3.524, *p* = 0.172, *W* = 0.09). The one-way repeated-measures ANOVA demonstrated an insignificant effect for time on uncertainty, *F*(1.528, 29.03) = 0.356, *p* = 0.647, partial η^2^ = 0.02. Thus, there was no significant decrease in parent-reported anxiety.

## 4. Discussion

The aim of this pilot study was to examine the effects of a 16-week school-based exercise program on the anxiety levels of children with moderate to severe symptoms of ASD in school and home settings. The results demonstrate that a 16-week exercise intervention can reduce anxiety in this population in the school setting. Moreover, the data show that greater reductions in anxiety were evident at week 16 in comparison to week 8.

As a result of the 16-week exercise program, teacher-rated total ASC-ASD scores and the performance anxiety, anxious arousal, and uncertainty subscales significantly improved among children with ASD. However, no significant improvements were evident for parent-reported levels of anxiety. The school-based exercise program resulted in large effect sizes on teacher-rated total ASC-ASD scores, performance anxiety, anxious arousal, and uncertainty, in addition to small effect sizes on separation anxiety, whereas parent-rated outcome measures demonstrated minimal to small effects.

There may be several explanations for the difference in results present between parents and teachers. First, it may be due to differences evident at baseline between the two raters. For total ASC-ASD, teachers reported a mean baseline score of 19.05 points. This score is just below the cut-off for significant anxious symptoms [38]. Meanwhile, parents reported a lower mean baseline score of 14 points. Previous research has shown that teachers tend to report higher levels of anxiety compared to parents [36], potentially because children with ASD may experience greater anxiety at school due to increased social and academic demands, in addition to the unpredictability of school environments [46,47,48]. Therefore, the positive effects of exercise on anxiety may be more profound in school settings.

Second, the difference in effects between teacher- and parent-reported anxiety levels may suggest that exercise has an immediate anxiolytic effect on children with ASD and thus is observable in the school setting, where the intervention is occurring, but not in the home setting. It is possible that the positive effects of the exercise sessions on anxiety levels may have been evident in the classroom after the session was complete but dissipated within a few hours prior to the child going home. Indeed, prior research has shown that physical activity has an immediate effect on anxiety, evident by the significant within-session reduction in self-reported anxiety and cortisol levels in Hillier et al. [23], but no long-term effects were present over 8 weeks. These results show that improvements in anxiety may be short-lived. Lastly, as teachers were more closely involved in the study and took part in the program, their responses may have been more prone to expectancy bias compared to parents.

In addition to Hiller et al. [23], only one other study assessed the effects of physical activity on anxiety in children with ASD. Golsefidi and Hashemi [24] found significant improvements in self-reported anxiety following 12 sessions of physical activity. The long-term effects noted by Golsefidi and Hashemi [24] and the present study, which are in contrast to the study of Hiller et al. [23], may be due to the higher frequency of sessions. Three sessions of exercise were implemented per week in the present study and three sessions of physical activity in Golsefidi and Hashemi’s [24] study, while Hiller et al. [23] implemented one session per week of physical activity, which did not lead to any long-term improvements in anxiety.

Very little attention has been given to quantifying the optimal dosage of exercise necessary to reduce anxiety in both neurotypical individuals and those with ASD [21]. The present study attempted to address this gap in the literature and demonstrated that greater improvements in anxiety were evident at week 16 compared to week 8. Teacher-reported anxiety levels significantly decreased from baseline to week 16, but not from baseline to week 8. A similar trend can also be seen in parent results, with greater improvements evident at week 16 compared to week 8, although these improvements were insignificant. Similarly, Hiller et al. [23] reported insignificant improvements in the anxiety levels of children with ASD following an 8-week physical activity intervention and recommended implementing a longer program. A longer exercise intervention may be more effective for children with moderate to severe ASD as some children may take some time to adapt to the program and the change in their routine.

As there is insufficient evidence to support the use of pharmacological interventions in children with ASD [13,14], along with CBT being potentially inappropriate to treat anxiety in children with severe symptoms of ASD, alternative treatments for anxiety are required for this population. This pilot study demonstrates the potential beneficial effects that exercise may have on anxiety in children with moderate to severe symptoms of ASD.

There are some limitations to the current study that should be noted for future studies. The inclusion of a control group would strengthen the study. Future studies should seek to test this study’s hypotheses with more rigorous experimental designs, including undertaking randomized controlled trials and controlling for confounders, such as the effects of the time of day on anxiety levels, increased socialization, and undertaking measurement of exercise intensity. As this study was unable to recruit female participants with ASD, future studies should examine if similar effects of physical activity on anxiety are found in a female population. The low response rate from parents and teachers is another limitation to the study. Not all parents and teachers completed the anxiety scale at all three timepoints. The attrition rate for the anxiety outcome measure was 42.55% for both teachers and parents. This is above previous intervention studies that consisted of children with ASD, which reported an attrition rate of less than 30% for questionnaire responses from parents. Future studies should also explore other methods to assess anxiety, including more objective measures such as cortisol levels. Validated interviews may be another option, which may increase response rates from parents and teachers.

## 5. Conclusions

The findings of this pilot study demonstrate that a 16-week school-based physical activity program can reduce anxiety in children with moderate to severe symptoms of ASD in school settings. Results demonstrate that 16 weeks, as opposed to eight, may be necessary to have a significant effect on in-school anxiety. Further investigations, utilizing experimental study designs, are required to refine the use of physical activity as a non-invasive, cost-efficient, convenient means of decreasing anxiety in children with ASD.

## Figures and Tables

**Table 1:** Sample of activities in the exercise program.

Activity	Aim	Description
Traffic lights	Increase heart rate and warming up the muscles	Different-colored cones will be assigned an action: green = run, yellow = walk, red = stop. Progression: Introduce more actions such as blue = jump, orange = skipping, purple = high knees
Throwing and catching	Develop throwing and catching skills, coordination	Throwing and catching a small light ball between two children. Progression: Change ball and distance. Different throwing techniques such as overhead, underhand. Aim for different targets such as hoops, baskets. Games such as basketball.
Jumping	Jumping and landing skills, lower limb strength, balance	Double leg jumping through hula hoops in a straight line. Progression: Increasing distance between hoops and number of hoops. Different jumping techniques: single leg, sideways. Introduce ladders and hurdles.
Kicking	Kicking skills, coordination, balance	Kicking a small light ball between two children. Progression: Increase the distance. Dribbling around cones. Taking shots at goals.
Volleyball	Striking with a hand, coordination, upper limb strength	Passing a beach ball between two children (volleyball set and bump technique). Progression: Using a volleyball. Hitting over a net.
Badminton	Striking with an implement, coordination, upper limb strength	Hitting a beach ball with a badminton racket between two children. Progression: Using a shuttle instead of beach ball. Hitting over a net. Using tennis rackets.
Stretches	Lower heart rate, flexibility	Butterfly stretch, toe touches, knee hugs, quadricep stretch, shoulder stretch.

**Table 2:** Characteristics of study participants.

Variables	Overall Sample (*n* = 24)		Teacher-Reported Sample (*n* = 20)		Parent-Reported Sample (*n* = 20)	
	M	SD	M	SD	M	SD
Age (years)	10.79	3.87	11.20	4.05	10.2	3.74
Moderate ASD (*n*, %)	6 (25%)		5		6	
Severe ASD (*n*, %)	18 (75%)		15		14	

**Table 3:** Results for the one-way repeated-measures ANOVA or Friedman test for teacher-reported ASC-ASD.

Variables	Baseline		Week 8		Week 16		*p*	% Change
	M	SD	M	SD	M	SD		Baseline–Week 16
Total ASC-ASD	19.05	12.08	14.55	12.48	6.8	5.77	<0.001	↓ 64.3%
Performance Anxiety	4.25	4.24	3	4.07	1.1	2.02	<0.001	↓ 74.12%
Anxious Arousal	3	2.43	1.75	2.22	0.55	0.95	<0.001	↓ 81.67%
Separation Anxiety	2.3	3.06	1.9	2.81	0.8	1.58	0.165	↓ 65.22%
Uncertainty	9.5	5.69	7.9	5.65	4.35	3.03	<0.001	↓ 54.21%

**Table 4:** Results for the one-way repeated-measures ANOVA or Friedman test for parent-reported ASC-ASD.

Variable	Baseline		Week 8		Week 16		*p*	% Change
	M	SD	M	SD	M	SD		Baseline–Week 16
Total ASC-ASD	14	9.41	13.1	7.46	12.75	8.64	0.911	↓ 8.93%
Performance Anxiety	1.2	1.85	1.3	2.34	1.15	2.08	0.879	↓ 4.17%
Anxious Arousal	2	2	1.35	1.39	1.35	1.81	0.41	↓ 32.5%
Separation Anxiety	1.65	1.79	2.05	1.71	1.9	2.1	0.172	↑ 3.03%
Uncertainty	9.15	6.01	8.4	4.8	8.35	5.6	0.647	↓ 8.74%

## Data Availability

Not applicable.
